# Another Whipple’s triad? Pericardial, myocardial and valvular disease in an unusual case presentation from a Canadian perspective

**DOI:** 10.1186/s12872-019-1257-2

**Published:** 2019-12-23

**Authors:** Christina S. Thornton, Yinong Wang, Martin Köebel, Kathryn Bernard, Tamara Burdz, Andrew Maitland, Jose G. Ferraz, Paul L. Beck, Andre Ferland

**Affiliations:** 1 Division of Respirology, Department of Medicine, University of Calgary, 3330 Hospital Drive NW, Calgary, AB T2N 4N1 Canada; 2 Department of Pathology and Laboratory Medicine, University of Calgary, Calgary, AB Canada; 3 Calgary Laboratory Services, Calgary, AB Canada; 4 National Microbiology Laboratory, Public Health Agency of Canada, Winnipeg, MB Canada; 5 Department of Medical Microbiology, University of Manitoba, Winnipeg, Canada; 6 Division of Cardiac Surgery, University of Calgary, Calgary, AB Canada; 7 Division of Gastroenterology and Hepatology, Department of Medicine, University of Calgary, Calgary, AB Canada; 8 Department of Critical Care Medicine, University of Calgary, Calgary, AB Canada

**Keywords:** Whipple’s disease, Culture-negative infective endocarditis

## Abstract

**Background:**

Whipple’s disease is a clinically relevant multi-system disorder that is often undiagnosed given its elusive nature. We present an atypical case of Whipple’s disease involving pan-valvular endocarditis and constrictive pericarditis, requiring cardiac intervention. A literature review was also performed assessing the prevalence of atypical cases of Whipple’s disease.

**Case presentation:**

A previously healthy 56-year-old male presented with a four-year history of congestive heart failure with weight loss and fatigue. Notably, he had absent gastrointestinal symptoms. He went on to develop pan-valvular endocarditis and constrictive pericarditis requiring urgent cardiac surgery. A clinical diagnosis of Whipple’s disease was suspected, prompting duodenal biopsy sampling which was unremarkable, Subsequently, *Tropheryma whipplei* was identified by 16S rDNA PCR on the cardiac valvular tissue. He underwent prolonged antibiotic therapy with recovery of symptoms.

**Conclusions:**

Our study reports the first known case of Whipple’s disease involving pan-valvular endocarditis and constrictive pericarditis. A literature review also highlights this presentation of atypical Whipple’s with limited gastrointestinal manifestations. Duodenal involvement was limited and the gold standard of biopsy was not contributory. We also highlight the Canadian epidemiology of the disease from 2012 to 2016 with an approximate 4% prevalence rate amongst submitted samples. Routine investigations for Whipple’s disease, including duodenal biopsy, in this case may have missed the diagnosis. A high degree of suspicion was critical for diagnosis of unusual manifestations of Whipple’s disease.

## Background

Whipple’s disease is a rare disease that classically afflicts middle-aged white men with an annual incidence of > 1/1,000,000. The prototypical clinical presentation of Whipple’s includes arthralgia (87%), diarrhea (81%), weight loss (93%), lymphadenopathy (52%), neurologic symptoms (33%), fever (38%) and melanoderma (41%), but with 15% of individuals lacking these classics symptoms [1–3].

Cardiac manifestations involving Whipple’s disease are atypical but are often described as endocarditis, considered the most useful clinical finding [3, 4]. Several of the case reports involving endocarditis have a geographic predominance from France [5] to southern Germany [6]. It has been speculated that prevalence of *T. whipplei* is higher in these so-thought ‘endemic’ areas or perhaps clinical suspicion of Whipple’s is greater. As the bacterium is ubiquitous within the soil environment, there has been speculation of an oral infectious route [4]. Amongst healthy individuals, *T. whipplei* has been found in 1–11% of stool samples, and up to 26% of sewage plant workers [7]. Pericardial involvement, namely constriction, is a rare clinical finding in patients with Whipple’s disease. To our knowledge, this is the first report of confirmed Whipple’s disease with constrictive pericarditis in concordance with multi-valvular pancarditis.

## Case presentation

A 56-year-old Caucasian male presented with a diagnosis of recurrent heart failure. Previously, he was given a diagnosis of palindromic rheumatism based on a four-year history of lower extremity migratory joint pain and swelling that failed to respond to NSAIDs and hydroxychloroquine. During this time, he also developed unintentional weight loss, fatigue and cognitive impairment. Past medical history was significant for a renal cell carcinoma, treated with a left nephrectomy 4 years prior to presentation. The patient had a 45-pack year smoking history. He had not travelled abroad except for New Zealand and Hawaii 25 years ago.

The patient had been previously evaluated over 4 years by numerous services including rheumatology, hematology, cardiology and dermatology with an extensive work-up, including blood cultures that were negative on repeat occasions (Table 1). Eleven months prior to current presentation, the patient developed recurrent acute congestive heart failure exacerbation, requiring multiple admissions with treatment involving diuretics and chest tubes, providing limited short-term symptomatic relief.

**Table 1:** Results of Work-up Preceding Hospital Admission

Test (Units)	Results from Work-up (Reference Range)^a^
Hemoglobin (g/L)	80 (137–180)
Mean corpuscular volume (fL)	84 (82–100)
Platelets (10^9^/L)	215 (150–400)
Leukocyte Count (10^9^/L)	4.9 (4–11)
Absolute Neutrophil Count (10^9^/L)	3.8 (2–8)
Creatinine (umol/L)	88 (50–120)
Aspartate aminotransferase (U/L)	48 (8–40)
Alanine aminotransferase (U/L)	66 (1–60)
Bilirubin total (umol/L)	18 (0–24)
Complement, C3 (g/L)	0.8 (0.6–1.6)
Complement, C4 (g/L)	0.2 (0.1–0.4)
HIV	Negative
Brucella	Negative
Tularensis	Negative
Yersina	Negative
Toxoplasmosis	Negative
Bartonella	Negative
Syphilis	Negative
Cytomegalovirus	Negative
Hepatitis B	Negative
Hepatitis C	Negative
Epstein-Barr virus	Negative
*Mycobacterium tuberculosis*	Negative
IgG4	Negative
Extractable nucleic antigen	Negative
Neutrophil cytoplasmic antibodies	Negative
Rheumatoid factor	Negative
Anti-cyclic citrullinated peptide antibody (U/mL)	795 (< 5)
Serum protein electrophoresis	Normal
AM cortisol (nmol/L)	491 (170–500)
C-reactive peptide (mg/L)	72 (0–8)
CT Scan	Numerous non-specific lymphadenopathy. A PET CT scan showed diffuse bilateral axillary, inguinal, mediastinum, abdominal and pelvic lymphadenopathy with no FDG uptake
Bone marrow biopsy	Minimal monoclonal B cells
Axillary lymph node biopsy	Inflammation with non-caseating clusters of various cell types with reactive follicular hyperplasia with some features of dermatopathic lymphadenitis and focal granuloma formation
Skin biopsy	Melanoderma
Transthoracic Echocardiogram	Mild reduction in left ventricular systolic function with hypokinesis to the inferior basal and mid-segments. Mild right ventricle systolic dysfunction was noted with moderate pulmonary hypertension (RSVP 55 mmHg). No evidence of constrictive pericardium and no significant valvular disease

^a^Where applicable

On this presentation, the patient had symptoms consistent with an exacerbation of congestive heart failure. The patient stated he had lost approximately 40 pounds over 6 months. He denied any GI symptoms, fevers, chills or night sweats. Physical examination revealed the patient as pale, afebrile and hemodynamically stable. He was noted to be cachectic with a weight of fifty-one kilograms (BMI: 17.8). Pitting edema of both lower extremities to the proximal shin with hyperpigmentation was noted. As observed on the initial CT scan, there was diffuse lymphadenopathy and right epitrochlear lymphadenopathy in particular was noted. Jugular venous distension was noted to 8 cm above the sternal angle with a positive Kussmaul’s sign on inspiration. The patient had a 3/6 systolic ejection murder without a pericardial knock. There was no oculomasticatory myorhythmia or supranuculear vertical gaze palsy observed.

**Fig. 1:**
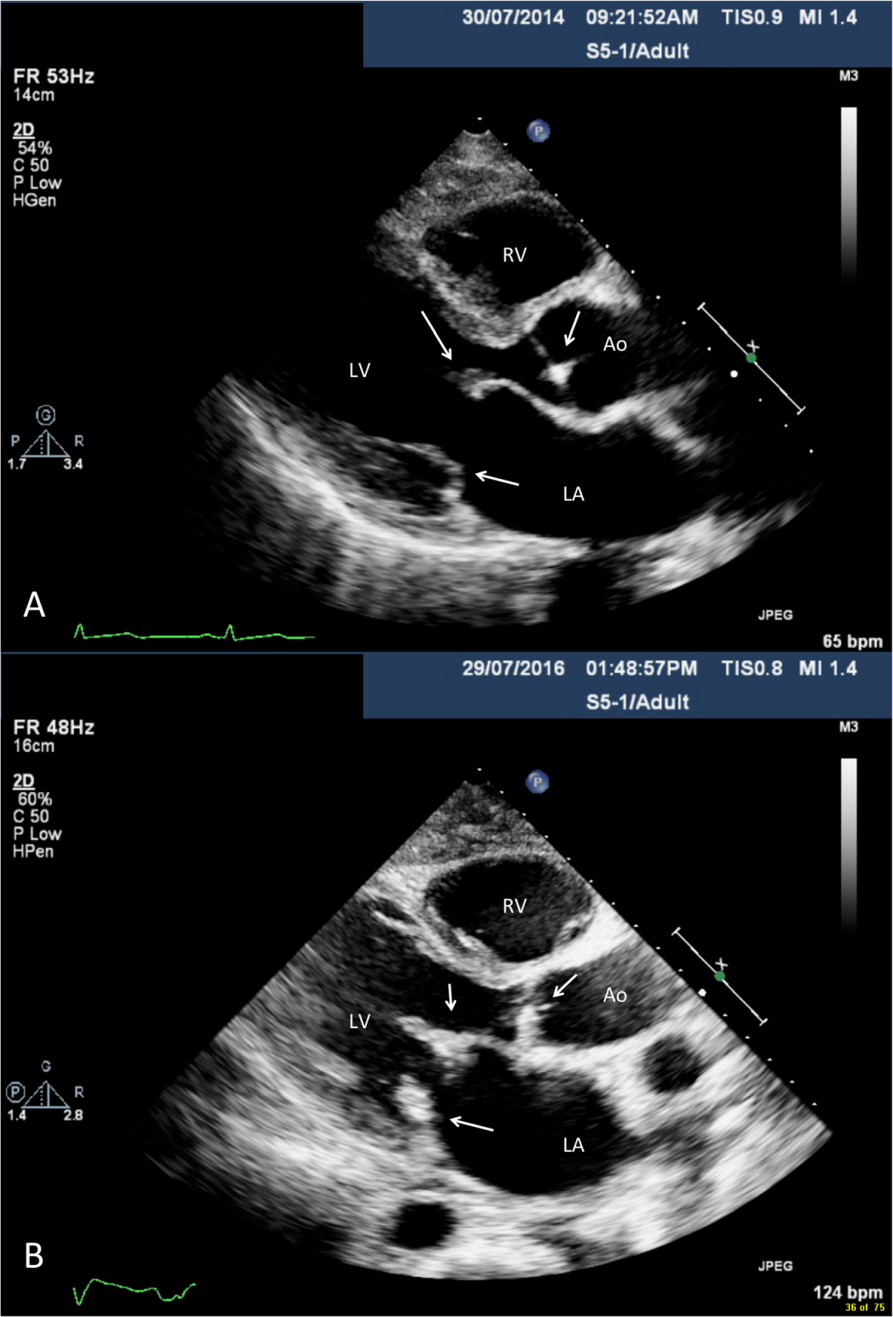
Parasternal long axis transthoracic echocardiogram findings in 2014 (**a**) and 2016 (**b**). Thickened mitral and aortic valve leaflets with diastolic doming of the mitral valve is depicted. Notably, in 2016 there was substantial interval thickening of the mitral and aortic valve leaflets

**Fig. 2:**
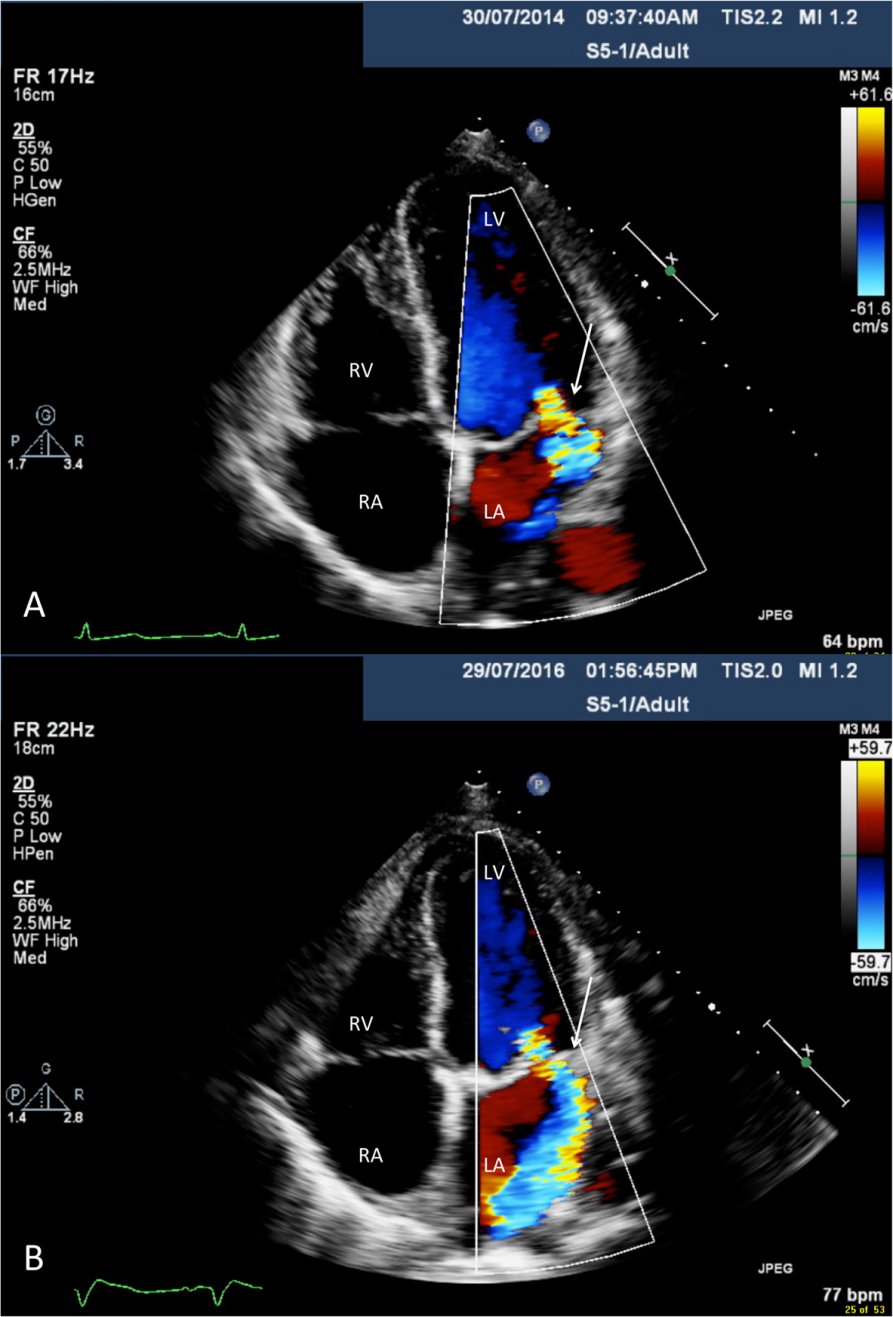
Apical four chamber view transthoracic echocardiogram findings in 2014 (**a**) and 2016 (**b**). Moderate-to-severe mitral regurgitation (MR) is noted with interval progression to severe MR in 2016

Transthoracic echocardiogram imaging was reviewed from 2014 to the 2016 (Figs. 1, 2, Additional files 1 and 2). In 2014 (Figs. 1a, 2a and Additional file 1), subtle tethering of the mitral valve was noted with thickening and cord calcification. The posterior leaflet had reduced excursion and the mitral valve apparatus was apically displaced. There was the observation of subtle diastolic doming over the anterior mitral leaflet. Progressive echocardiogram findings in 2016 (Figs. 1b, 2b and Additional file 2) showed the anterior mitral valve to be thickened with reduced excursion, the posterior leaflet to be fixed and cords with shortening. Overall, this was in keeping with pseudoprolapse. Over the aortic valve, there was systolic doming of the right coronary cusp and calcification from the commissural aspect of the aortic valve with sparing of the base. Notably, there was the presence septal shuttering in early diastole with early inspiratory septal bounce/shift, indicative of abnormal heart and lung interaction. Overall, this was suggestive of early constrictive physiology.

A repeat transthoracic echocardiogram on admission showed numerous interval changes including; a reduction in left ventricle systolic function (ejection fraction of 48%), severe mitral regurgitation, moderate tricuspid regurgitation, moderate aortic regurgitation and worsening pulmonary hypertension (RSVP 78 mmHg). Calcific aortic valve changes were noted that were felt to be post-inflammatory as opposed to degenerative changes. The mitral valve had an unusual appearance suggestive of prior valvulitis, in particular rheumatic in nature. The pericardium was thickened with features of exaggerated heart and lung interactions, consistent with constrictive physiology. Based on these findings, pericardial stripping and valvular surgery were proposed.

Perioperative findings consisted of thick pericardium and densely adherent to the surrounding anatomy. The mitral valve was grossly abnormal with fibrosis, in keeping with a rheumatic type process and was subsequently replaced with a bioprosthetic valve. The tricuspid valve had thickened leaflets with slightly thickened papillary muscle and was repaired with a ring annuloplasty. The thickened aortic valve was addressed with primary repair.

**Fig. 3:**
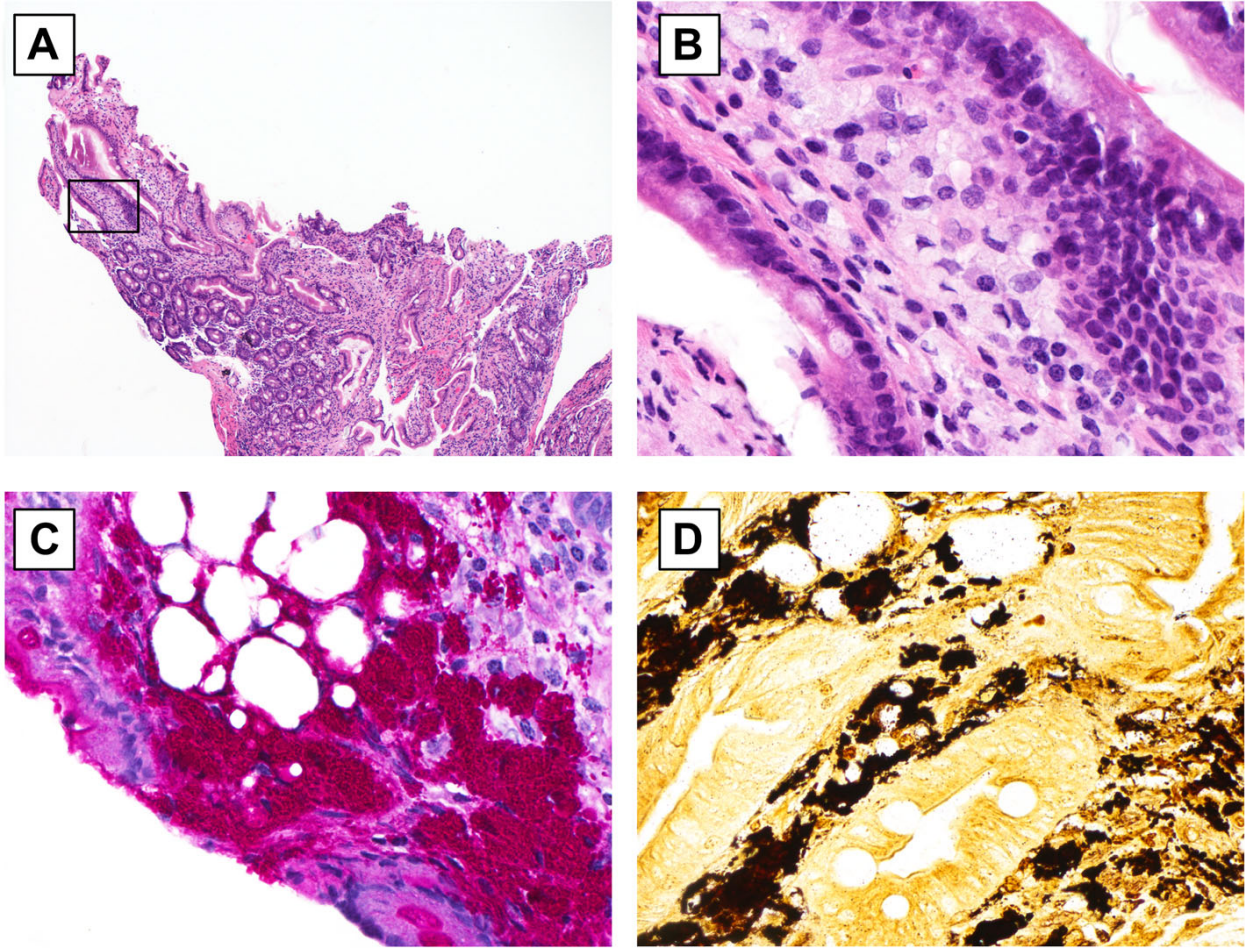
Duodenal biopsy hematoxylin eosin stain (low power, 4x) with reactive appearance and focal lamina propria infiltrate of foamy macrophages (rectangle). Note, a massive infiltration of lamina propria by foamy macrophages is absent. **b** Duodenal biopsy hematoxylin eosin stain (high power, 40x) from rectangle (**a**) shows foamy macrophages. **c** Duodenal biopsy periodic acid-Shiff stain (high power, 40x) highlights intensily PAS+ Tropheryma whippelii with macrophages. The lamina propria also contains small foci of fat. **d** Duodenal biopsy Warthin Starry stain (high power, 40x) highlights Tropheryma whippelii with macrophages

**Fig. 4:**
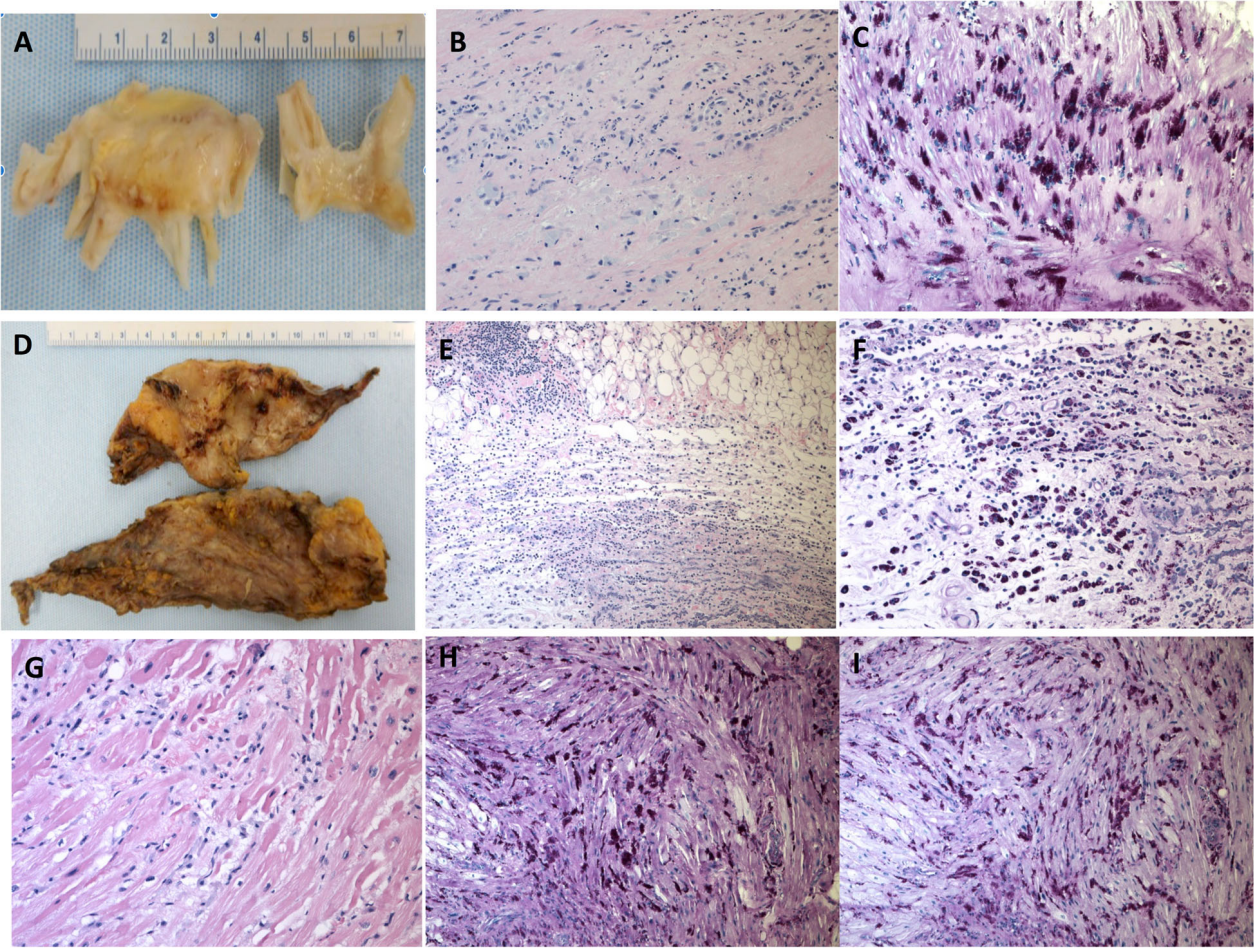
Gross and histopathology findings: Fibrotic thickening of mitral valve (**a**) and pericardium (**d**). Microscopic appearance of mitral valve (**b**), hematoxylin-eosin, original magnification × 100), atrial myocardium (**g**), hematoxylin-eosin, original magnification × 200), and pericardium (**e**), hematoxylin-eosin, original magnification × 100), inflammatory infiltration with numerous macrophages, the myocardium also shows myocyte damage. The cytoplasm of macrophages is filled with periodic acid-Schiff-positive material (**c**, **f**, and **h**), original magnification × 200)

The above constellation of symptoms, in the context with prior work-up as unremarkable, lent to a strong clinical suspicion of an atypical presentation of Whipple’s disease. Two days post-op, duodenal biopsies were obtained (Fig. 3a, b), and surprisingly were not consistent with Whipple’s disease. On the request of the clinician, the cardiac valves and pericardium pathology specimens were examined for PAS staining and PCR. The cardiac specimens demonstrated numerous foamy macrophages filled with PAS positive material compatible with Whipple’s disease (Fig. 4). *Tropheryma whipplei* was identified by direct 16S rDNA PCR on the mitral valve with > 99% sequence match. The pathological changes were consistent with active endocarditis, myocarditis, and pericarditis, caused by *T. whipplei*. The patient started treatment with IV Penicillin G for 14 days and subsequently maintained regimen of oral sulfamethoxazole-trimethoprim for long-term treatment. Six months following diagnosis, the patient has had no complications and is clinically improving.

## Discussion and conclusions

The diagnosis of *T. whipplei* valvular involvement is elusive as there is the absence of clear diagnostic criteria. Often the diagnosis hinges on 16S rDNA PCR following surgical removal of valvular tissue coinciding with high clinical suspicion from physician vigilance. Currently, limited serologic assays only can distinguish between classic Whipple disease and asymptomatic gastrointestinal carriers.

The diagnostic criterion for Whipple’s disease includes two of three tests to be positive from PAS staining, PCR or immunohistochemistry [8]. The gold standard tissue is considered to be histology from small bowel biopsies, which classically present as PAS-positive foamy macrophages within the lamina propria [9]. However, PAS-positive macrophages are not specific and have been observed in patients with intestinal infections (*Mycobacterium avium* complex, *Rhodococcus*. sp., *Bacillus cereus*, *Corynebacterium* spp., and *Histoplasma* spp.,), Crohn’s disease, histiocytosis and Waldenstrom’s macroglobulinemia [9–11].

We also sought to assess the epidemiology of Whipple’s disease in Canada, which previously was unknown. A consortium of data collected from the National Microbiology Laboratory in Canada was done from 2012 to 2016 and highlighted in Table 2. Notably, only 4% of samples were positive from those collected; of which 13.5% were elucidated from the gold standard of duodenal biopsy, with 86.2% of other tissue types being found to be positive for *T. whipplei*. A limitation of this should be noted in that multiple samples were present from the same patient. However, this highlights the diagnostic utility of other samples apart from the previously denoted ‘gold standard’ of duodenum.

**Table 2:** Total number of samples submitted for *Tropheryma whipplei* PCR testing, number of positive samples, and percent positivity from 2012 to 2016 in Canada

Sample type	2012			2013			2014			2015			2016			Totals		
	No. Samples	No. Pos	% Pos	No. Samples	No. Pos	% Pos	No. Samples	No. Pos	% Pos	No. Samples	No. Pos	% Pos	No. Samples	No. Pos	% Pos	No. Samples	# Pos	% Pos
GI tract*	30	7	23.33	26	2	7.69	32	3	9.38	42	5	11.90	32	5	15.63	162	22	13.58
Cardiac	3	0	0.00	2	0	0.00	3	1	33.33	5	1	20.00	10	1	10.00	23	3	13.04
Lymph node	4	1	25.00	6	1	16.67	3	0	0.00	0	0	0.00	7	0	0.00	20	2	10.00
Brain	2	0	0.00	1	0	0.00	1	0	0.00	2	0	0.00	6	0	0.00	12	0	0.00
Other tissue	9	0	0.00	6	1	16.67	6	0	0.00	8	1	12.50	16	1	6.25	45	3	6.67
Blood	57	0	0.00	85	2	2.35	102	3	2.94	101	2	1.98	104	1	0.96	449	8	1.78
CSF	41	0	0.00	63	2	3.17	54	0	0.00	76	2	2.63	102	0	0.00	336	4	1.19
Other fluid	4	0	0.00	1	0	0.00	0	0	0.00	0	0	0.00	5	0	0.00	10	0	0.00
Total	150	8	5.33	190	8	4.21	201	7	3.48	234	11	4.70	282	8	2.84	1057	42	3.97
# Positive patients**	6			6			5			9			8					

*GI tract includes fresh or paraffin embedded tissue samples from small and large intestine and samples labelled as “gastric”

**Multiple positive sample types may be submitted from the same patient

Our patient presented with heart failure due to pericardial constriction with multiple cardiac valve involvement in the context of a systemic process including cachexia and neurological impairment with reduced cognition. He did not display classical Whipple’s symptoms including gastrointestinal manifestations and had few PAS-positive macrophages within the intestinal wall. Given the current diagnostic guidelines, this may substantially underrepresent the true incidence of Whipple’s disease in the absence of overriding strong clinical suspicion. The accurate clinical evaluation of the patient described in this case is likely multifactorial and may represent improved modern medical care to bridge to surgery, improved current diagnostic imaging and high clinical acumen. PAS staining is not routinely performed on cardiac specimens at our institution. Furthermore, this patient represents the first description in the literature of constrictive pericarditis with pan-valvular findings. Constrictive pericarditis, involving cardiac valvular pathology, in the absence of common etiologies should promote clinical suspicion as a manifestation of Whipple’s disease even in the absence of gastrointestinal manifestations.

This case further highlights as a key example of blood culture-negative endocarditis (BCNE). BCNE are thought to account for 2.5–70% of all cases of endocarditis based upon standard culture-dependent laboratory techniques, with a differential diagnosis including both infectious and non-infectious etiologies (Table 3) [13]. Among culture-negative endocarditis, *T. whipplei* was found to be the fourth most common cause as demonstrated by both histological and molecular techniques [14]. A recent study from France demonstrated that by adding real-time PCR assays in patients with BNCE, not only did the diagnostic efficiency improve by 24.3%, but detection of atypical organisms including *T. whipplei* also increased, thus improving diagnostic certainty [15]. In Canada, epidemiological rates of BCNE are currently unknown, but given the prevalence from prior case series from other countries is likely underreported.

**Table 3:** Differential Diagnosis for Blood Culture Negative Endocarditis by Etiology^a^

Infectious	Non-Infectious
⦁ Bartonella species.	⦁ Antiphospholipid syndrome
⦁ *Brucella melitensis*	⦁ Atrial myxoma
⦁ Chlamydia species	⦁ Marantic endocarditis
⦁ Corynebacterium species	⦁ Libman-Sachs endocarditis
⦁ *Coxiella burnetii*	⦁ Mural thrombi
⦁ Enterobacteriaceae species	⦁ Lambl’s excrescences
⦁ Fungal species (mostly Candida)	⦁ Cholesterol emboli
⦁ HACEK bacteria	⦁ Vasculitis (Behcet’s)
⦁ Legionella species	⦁ Paradoxical emboli
⦁ Mycoplasma species	⦁ Left atrial thrombi
⦁ Staphylococcus species	
⦁ Streptococcus species	
⦁ *Tropheryma whipplei*	

^a^Modified from Fournier, et al. [12]

Ultimately, as this case highlights, repeated negative blood cultures should prompt the clinician to further evaluate atypical causes of BCNE, including Whipple’s disease. This case further reinforces prior studies in which addition of molecular studies may aid in diagnostic certainty by increasing positive yield. Further research is required in order to optimize medical care and highlighting a likely under-diagnosed disease; both with regards to Whipple’s disease and BCNE.

## Supplementary information

**Additional file 1.** Transthoracic echocardiography in 2014 revealed thickened mitral and aortic valve leaflets with diastolic doming of the mitral valve across parasternal long axis view, apical four chamber, apical four chamber mitral valve with colour, parasternal short axis aortic valve and parasternal short axis mitral valve views.

**Additional file 2.** Transthoracic echocardiography in 2016 revealed interval deterioration with further thickening of the mitral and aortic valve leaflets with diastolic doming of the mitral valve across parasternal long axis view, apical four chamber, apical four chamber mitral valve with colour, parasternal short axis aortic valve and parasternal short axis mitral valve views. The final clip depicts septal shuttering in early diastole, suggestive of early constrictive physiology.

## Data Availability

Not applicable.

## Abbreviations

BCNE: Blood-culture negative endocarditis
BMI: Body mass index
GI: Gastrointestinal
NSAID: Non-steroidal anti-inflammatory drugs
PAS: Periodic acid-Schiff stain
PCR: Polymerase chain reaction
rDNA: Ribosomal deoxyribonucleic acid
RVSP: Right ventricular systolic pressure
